# USP7 overexpression predicts a poor prognosis in lung squamous cell carcinoma and large cell carcinoma

**DOI:** 10.1007/s13277-014-2773-4

**Published:** 2014-12-18

**Authors:** Guang-Yin Zhao, Zong-Wu Lin, Chun-Lai Lu, Jie Gu, Yun-Feng Yuan, Feng-Kai Xu, Rong-Hua Liu, Di Ge, Jian-Yong Ding

**Affiliations:** 1 Department of Thoracic Surgery, The Affiliated Zhongshan Hospital of Fudan University, 200032 Shanghai, People’s Republic of China; 2 Department of Immunology, Shanghai Medical College, Key Laboratory of Molecular Medicine of Ministry of Education, Fudan University, 200032 Shanghai, People’s Republic of China

**Keywords:** Lung cancer, USP7, Prognosis, Apoptosis

## Abstract

In non-small cell lung cancer (NSCLC), both USP7 expression and p53 gene status were reported to be an indicator of poor prognosis in adenocarcinoma patients; however, its roles and mechanisms in lung squamous cell carcinoma and large cell carcinoma need to be clarified. The USP7 expression was examined in NSCLC tumors (excluding adenocarcinoma), their corresponding non-tumorous tissues, and NSCLC cells. Then, the prognostic role of USP7 was analyzed in 110 NSCLC samples (excluding the adenocarcinoma). Finally, the roles and mechanisms of USP7 in the proliferation, metastasis, and invasion of a NSCLC cell were assessed using a specific vshRNA. The USP7 expression was higher in NSCLC tissues compared to non-tumorous samples, accordingly, the high level of USP7 was detected in NSCLC cell lines compared with HBE cell. After the USP7 downregulation, the H460 cells exhibited decreased metastasis/invasion in vitro and in vivo. The preliminary mechanism study indicated overexpression of USP7 might regulate the p53-MDM2 pathway by inducing the MDM2 de-ubiquitination and subsequent stabilization, which resulted in the upregulation of the Bad phosphorylation. Additionally, we also found that USP7 might induce cell epithelial-mesenchymal transition to enhance the cell invasive ability. Clinically, USP7 overexpression significantly correlated with malignant phenotype. Furthermore, the 5-year overall survival in patients with USP7^low^ was higher than that of USP7^high^. Multivariate analysis showed USP7 overexpression was an independent prognostic marker for these cancers. USP7 overexpression may regulate the survival and invasive properties of squamous cell carcinoma and large cell carcinoma cells, and may serve as a molecular target.

**Electronic supplementary material:**

The online version of this article (doi:10.1007/s13277-014-2773-4) contains supplementary material, which is available to authorized users.

## Introduction

Lung cancer is a highly aggressive malignancy and the leading cause of cancer-related mortality worldwide. Non-small cell lung cancer (NSCLC) accounts for nearly 85 % of lung cancer cases. Despite great improvements in diagnostic technology and the introduction of new therapeutic agents in recent years, the 5-year survival rate of NSCLC patients remains dismal due to NSCLC cell invasion and metastasis prior to treatment [1, 2]. Therefore, it is essential to identify new sensitive and specific molecular markers for the early detection and therapeutic targeting of NSCLC.

USP7 (also known as herpesvirus-associated ubiquitin-specific protease, HAUSP) was originally identified as a herpesvirus-associated cellular factor and subsequently shown to deubiquitinate and stabilize p53 [3]. Previous studies showed that USP7 can target several key regulatory proteins, including tumor suppressors, DNA repair proteins, immune responders, viral proteins, epigenetic modulators [4], such as the herpes simplex virus type 1 (HSV1) Vmw110 interacting protein, Epstein-Barr nuclear antigen 1 (EBNA1), p53 regulator murine double minute (MDM2), PTEN, INK4a, TSPYL5, Ataxin-1, and the transcription factors FOXO4, and REST. USP7 deubiquitinase activity on these substrates can enhance protein stability and alter the subcellular localization of targets. Recent studies have shown that the overexpression of USP7 in prostate cancer correlates with tumor aggressiveness and that the inhibition of USP7 can induce the apoptosis of multiple myeloma (MM) cells resistant to conventional and bortezomib therapies [5, 6]. Thus, contrary to initial reports, high levels of USP7 may promote tumor progression.

USP7 knockout mice die during early embryonic development compared to wild-type mice, and USP7 knockout embryos show p53 activation and cell growth arrest [7]. In NSCLC, Masuya et al. reported that USP7 mRNA expression was significantly lower in adenocarcinomas than in squamous cell carcinomas, and 45.0 % patients showed reduced expression of USP7 and the reduction of USP7 gene expression was reported to play an important role in adenocarcinomas through p53-dependent pathways [8], However, the detailed roles of USP7 in NSCLC should be performed to clarify, for example, the expression of USP7 in NSCLC compared with the corresponding non-tumorous tissues and its roles and mechanisms in lung squamous cell carcinomas and large cell carcinoma.

Here, we found USP7 expressed high in NSCLC tissues (excluding the adenocarcinoma tissues) compared with adjacent non-tumorous tissues. Furthermore, the increased USP7 expression is positively correlated with cancer stage, lymph node metastasis, and tumor size. Importantly, high level of USP7 expression was found to be associated with the poor outcome of lung cancer patients (excluding the adenocarcinoma patients). The knockdown of USP7 in NSCLC cells impaired cell invasion and motility, and tumor formation, and induced cell apoptosis. Thus, USP7 might be used as a prognostic factor and therapeutic target in treating lung squamous cell carcinomas and large cell carcinoma.

## Materials and methods

### Animals

Male athymic BALB/c nude mice (4 weeks old, Shanghai Institute of Material Medicine, Chinese Academy of Science) were raised in specific pathogen-free conditions. All animal experiments were done in accordance with a protocol approved by the Shanghai Medical Experimental Animal Care Commission.

### Cell lines and cell transfection

The cell lines A549, H460, H1299, H1355, 95-D, and 95-C were purchased from the Institute of Biochemistry and Cell Biology at the Chinese Academy of Science. The cells were maintained in Dulbecco Modified Eagle medium (DMEM) or RPMI 1640 supplemented with 10 % fetal bovine serum and 1 % penicillin-streptomycin.

The pGMLV-SC5RNAi lentiviral vectors were purchased from Shanghai Genomeditech Co. Ltd, and the vshRNA were constructed and synthesized by Shanghai Genomeditech Co. Ltd. The lentiviral vector was transfected into cells according to the manufacturer’s instructions. The sequence strands of vshRNAs are shown in Supplementary Table 1.

### Patients and follow-up

Archival specimens were obtained from 110 patients of lung cancer (including squamous cell carcinomas and large cell carcinoma) at Zhongshan Hospital (Shanghai, People’s Republic of China) in 2005 with informed consent. All patients who underwent curative resection for NSCLC were included in our study. All patients underwent standard lobectomy and mediastinal lymph node dissection. Paraffin blocks were only selected if suitable formalin-fixed, paraffin-embedded tissue and complete clinicopathologic and follow-up data for the patients were available. Tumor stage was determined according to the tumor/lymph node/metastasis (TNM) classification using the seventh edition of the International Union Against Cancer Staging Manual. Pathologic classification was based on World Health Organization criteria. Follow-up was completed in July 2010 and the median follow-up was 43 months (range, 1–66 months). Overall survival (OS) was defined as the interval between surgery and death or between surgery and the last observation for surviving patients. The data were censored at the last follow-up for living patients. Ethical approval was obtained from the Zhongshan Hospital Research Ethics Committee.

### Real-time quantitative reverse transcription PCR and Western blot analysis

Total RNA was extracted using the TRI Reagent (Sigma-Aldrich, St. Louis, MO, USA) according to the manufacturer’s protocol. Complementary DNA was synthesized from 2 μg of total RNA using random hexamers (Proligo, Boulder, CO) and SuperScript III Reverse Transcriptase (Invitrogen, Carlsbad, CA). RT-PCR was carried out on a panel of cell lines and tumor samples. The real-time quantitative reverse transcription PCR (qRT-PCR) conditions were as follows: 1 min at 95 °C, denaturation at 95 °C for 5 s, annealing at 60 °C for 30 s, and extension at 72 °C for 60 s. As follows are the primers of USP7: 5′-GTTGTTGGAGCGATTACAAGA-3′ and 5′-AAACTGGTCCTCTGCGACTATC-3′; β-actin: 5′-GGCATCCTGGGCTACACTGA-3′ and 5′-GTGGTCGTTGAGGGCAATG-3′. Relative expression among samples was calculated by the comparative CT method. All experiments were performed in triplicate.

Thirty micrograms of total extract protein was run on SDS-polyacrylamide gel electrophoresis gels, transferred onto polyvinylidene difluoride membranes, and incubated with the corresponding antibodies. The membranes were developed with the enhanced chemiluminescence method (Pierce, Rockford, IL, USA). The primary antibodies used were USP7 (1:1,000, Abcam, UK). β-Actin protein detection using β-actin antibodies (1:2,000, Beyotime, China) was used as an internal control. All experiments were performed in triplicate.

### Proliferation assay, monolayer colony formation assay, and cell apoptosis assay

According to the manufacturer’s instructions, CCK8 (Beyotime, China) was used to measure the proliferation of H460-vshUSP7, A549-vshUSP7, and control cells. The specific process was previously described [9].

For the colony formation assay, cells were planted into three 6-cm cell culture dishes (1,000 cells per dish) and incubated for 12 days. Plates were washed with PBS then stained with Giemsa. Colonies with more than 50 cells were counted.

According to the manufacturer’s instructions, an Annexin V-PE Apoptosis Detection Kit (KeyGEN Biotech, China) was used to measure apoptosis in the H460, H460-vshUSP7, A549, and A549-vshUSP7 cells. First, 2 × 10^5^ cells were collected and then washed twice with PBS. Cells were then resuspended in 500 μl binding buffer and transferred to the EP tube. After adding 1 μl Annexin V-PE and mixing at room temperature, samples were placed in the dark for 15 min. Cell apoptosis was then detected by flow cytometry. Experiments were repeated in triplicate.

### Cell invasion assays

Cell invasion assays were performed using 24-well transwell plates (Corning, NY, USA) precoated with Matrigel (Falcon354480; BD Biosciences, Franklin Lakes, NJ, USA). The lower chambers were filled with growth media and the upper chambers were filled with media and 1 % FBS. A549 cells were seeded in the upper chamber of inserts at a density of 1 × 10^5^ cells per well. After 24 h, the inserts were removed and washed, the upper Matrigel layer removed, and the membrane and Matrigel were fixed in 4 % paraformaldehyde and stained with Giemsa. All experiments were performed in triplicate.

### In vivo animal studies

H460-shUSP7 cells (1 × 10^6^) and H460-control (1 × 10^6^) cells were subcutaneously implanted into either posterior flank of the same nude mouse. Tumor growth was monitored every week, and after 20 days, the tumor volumes were measured and statistically analyzed.

### Tissue microarray analysis and immunohistochemistry

TMAs were constructed as previously described [10], and immunohistochemistry protocols were described elsewhere [9]. USP7 antibodies (Abcam, UK) were used to detect USP7 protein expression. The intensity of positive staining was measured as described [10]. The intensity of USP7 protein expression was classified into high expression and low expression according to a cutoff value of 40 %.

### Statistical analysis

Statistical analyses were performed with SPSS 16.0 software (SPSS, Chicago, IL). Cumulative survival time was calculated by the Kaplan-Meier method and analyzed by the log-rank test. Cox’s proportional hazards regression model was used to analyze the independent prognostic factors. For the comparison of individual variables, *t* tests, *χ* ^2^ tests, Fisher’s exact tests, and Spearman coefficients tests were carried out as appropriate. Statistical significance was determined for two-tailed tests at *p* < 0.05.

## Results

### USP7 is frequently upregulated in NSCLCs and positively correlates with poor NSCLC prognosis

**Fig. 1:**
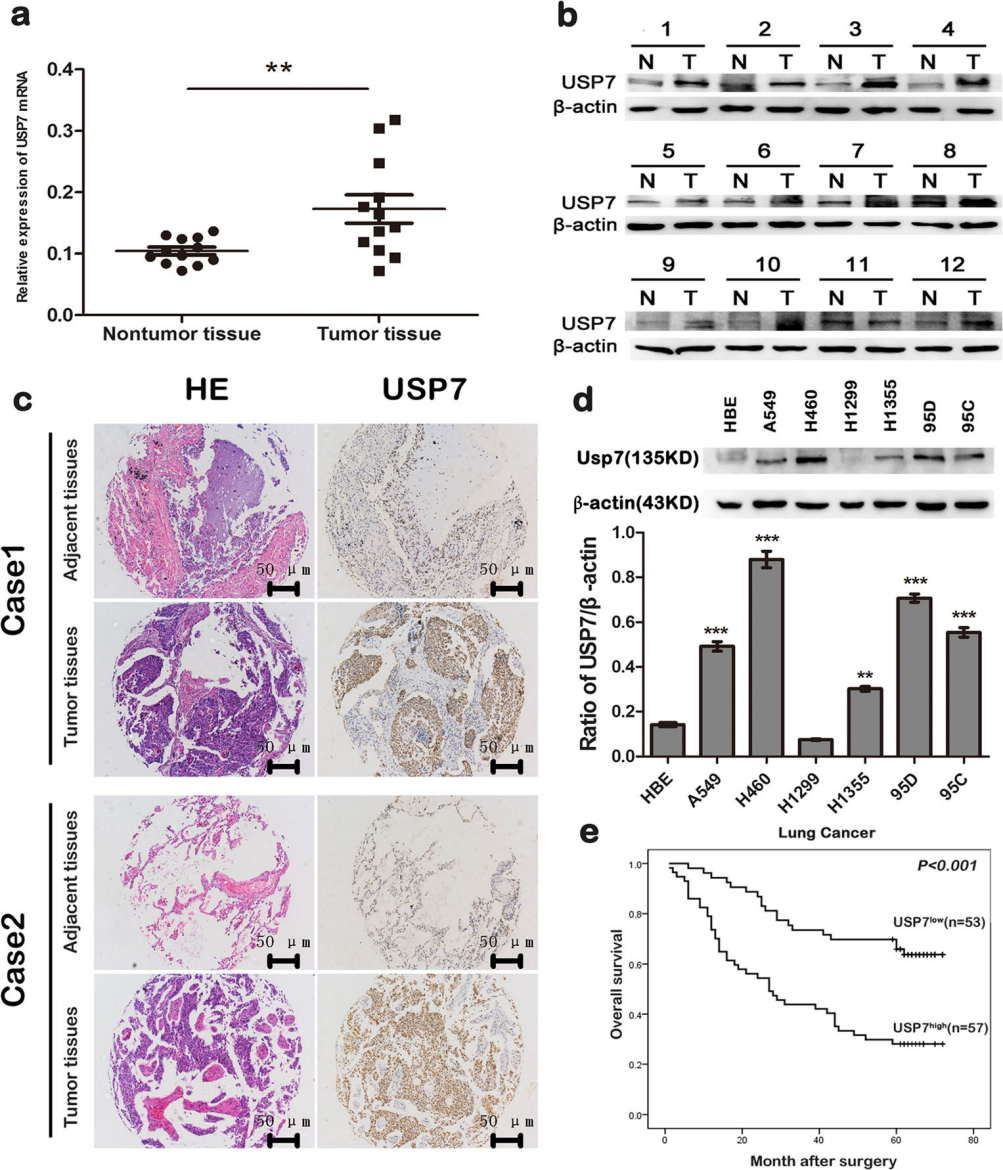
USP7 is frequently upregulated in NSCLCs and positively correlates with poor NSCLC prognosis. **a**, **b** USP7 mRNA and protein expression in paracancerous and cancer tissues from squamous cell carcinoma and large cell carcinoma patients; **c** USP7 protein expression in paracancerous and cancer tissues from 110 NSCLC patients determined by IHC (excluding the adenocarcinoma); **d** USP7 expression in six NSCLC cell lines and one normal bronchial epithelial cell line; **e** prognostic significance of USP7 expression in squamous cell carcinoma and large cell carcinoma patients assessed by Kaplan-Meier survival estimates

To investigate the USP7 expression in NSCLC tissues, we first detected USP7 expression in 12 pairs of fresh primary NSCLC tumors (excluding the adenocarcinoma tissues) and their corresponding non-tumorous tissues by qRT-PCR and Western blot analysis. USP7 upregulation was detected in 11 out of 12 tumor tissues compared with their normal counterparts (Fig. 1a, b). Then, we further investigated the USP7 protein expression in 110 primary squamous cell carcinoma and large cell carcinoma and their adjacent normal lung tissues by IHC using tissue microarrays (TMA). USP7 upregulation was detected in 57 out of 110 (51.82 %) tumor tissues compared with their adjacent non-tumorous tissues (Fig. 1c).

Furthermore, we investigated USP7 expression in A549, H460, H1299, H1355, 95D, and 95C NSCLC cell lines compared with the immortalized lung epithelial cell line HBE by Western blot analysis. NSCLC cells expressed high levels of USP7 compared with immortalized lung epithelial cells (Fig. 1d).

To investigate whether USP7 overexpression correlates with clinical squamous cell carcinoma and large cell carcinoma progression, we analyzed USP7 expression in the cohort of 110 squamous cell carcinoma and large cell carcinoma patients. USP7 expression positively correlated with a more advanced tumor stage (*p* < 0.001), lymph node metastasis (*p* = 0.006), and bigger tumor size (*p* = 0.038). However, there were no significant relationships between USP7 and other factors, such as age, gender, smoking status, and differentiation (Table 1).

**Table 1:** Clinicopathologic variables in 110 NSCLC patients

Variables	No. of patient	USP7 expression		*p* value
		low	high	
Age				
<51	27	15	12	0.377
≥51	83	38	45	
Gender				
Male	60	28	32	0.728
Female	50	25	25	
Smoker status				
Smoker	31	14	17	0.691
Non-smoker	79	39	40	
Tumor stage				
I–II	77	46	31	<0.001
III–IV	33	7	26	
Lymph node metastasis				
Yes	46	15	31	0.006
No	64	38	26	
Tumor size				
<3 cm	49	29	20	0.038
≥3 cm	61	24	37	
Differentiation				
Well/moderate	74	38	36	0.340
Poor	36	15	21	

We next divided the 110 patients into two groups: those with a high expression of USP7 (USP7^high^) and those with a low expression of USP7 (USP7^low^). Univariate analysis revealed that tumor size (≥3 cm, *p* < 0.001), lymph node metastasis (*p* < 0.001), advanced tumor stage (*p* < 0.001), and high USP7 expression (*p* < 0.001) were associated with OS (Table 2). We subsequently analyzed the impact of USP7 expression on the survival of NSCLC patients. We found that the 5-year OS in the USP7^low^ group was significantly higher than the USP7^high^ group (63.7 versus 28.1 %). The multivariate Cox proportional hazards model was used for further analysis and high USP7 expression was an independent prognostic predictor for OS (*p* = 0.007) (Table 2, Fig. 1e).

**Table 2:** Univariate and multivariate analysis of factors associated with OS

Variables	Univariate analysis^a^	*p* value	Multivariate analysis^b^	*p* value
	HR		HR	
Age (≥51 versus <51)	1.698 (0.882–3.267)	0.113		
Gender (female versus male)	0.610 (0.362–1.028)	0.063		
Smoking status (non-smoker versus smoker)	0.695 (0.403–1.198)	0.190		
Differentiation (poor versus well/moderate)	1.787 (1.064–3.002)	0.028	1.426 (0.845–2.408)	0.184
Tumor size (≥3 cm versus <3 cm)	2.854 (1.622–5.022)	<0.001	2.319 (1.297–4.147)	0.005
Lymph node–metastasis (yes versus no)	3.285 (1.952–5.530)	<0.001	2.467 (1.333–4.600)	0.004
Tumor stage (III–IV versus I–II)	2.864 (1.708–4.802)	<0.001	1.031 (0.535–1.987)	0.927
USP7 expression (high versus low)	2.989 (1.730–5.166)	<0.001	2.244 (1.247–4.040)	0.007

*OS* overall survival, *95 % CI* 95 % confidence interval

^a^Variables were adopted for their prognostic significance (*p* < 0.05) in univariate analysis using forward, stepwise selection (forward likelihood ratio)

^b^A Cox proportional hazards regression model was used for multivariate analysis

### Large cell carcinoma cell tumorigenesis is inhibited by USP7 knockdown in vitro and in vivo

**Fig. 2:**
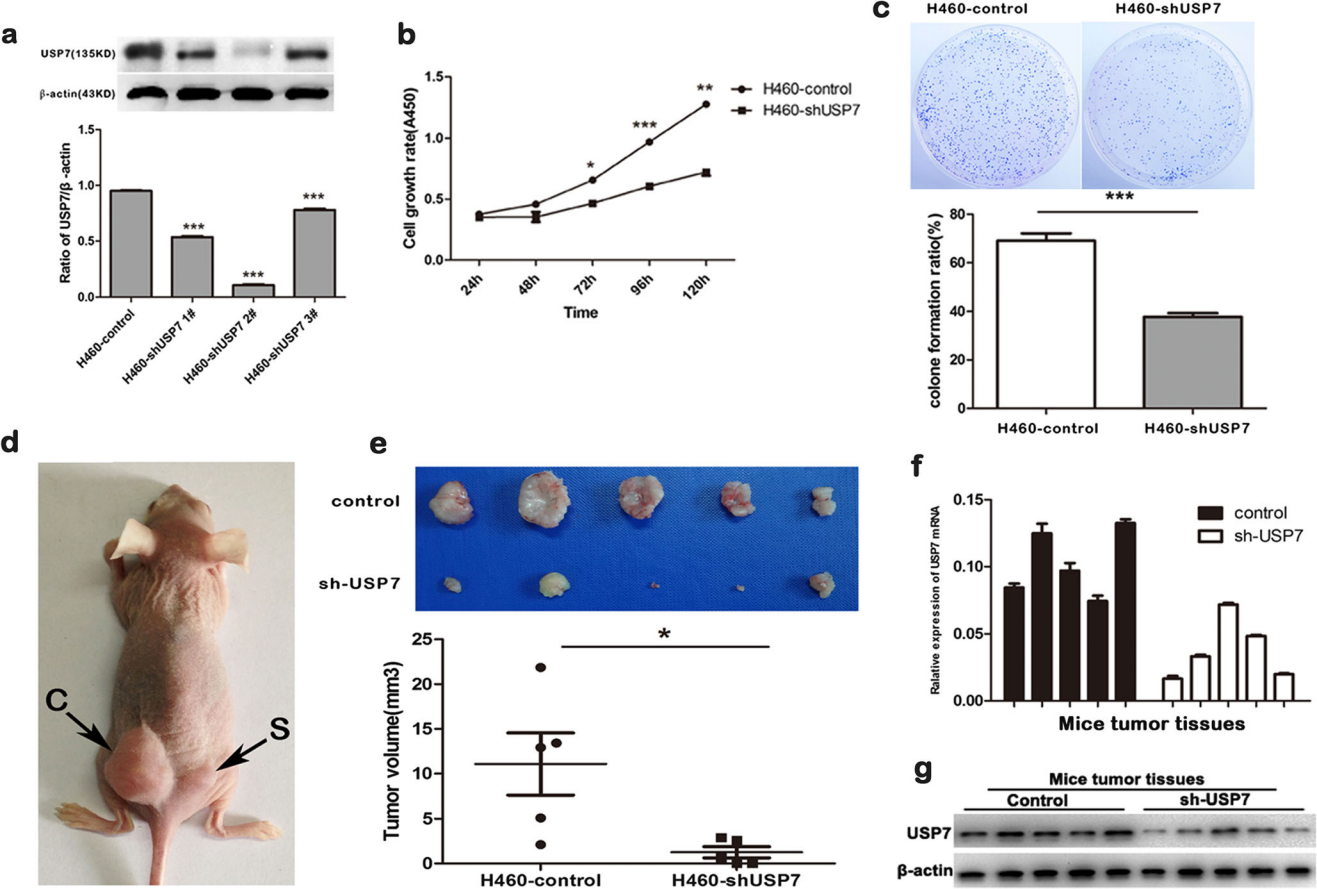
Large cell carcinoma cell tumorigenesis is inhibited by USP7 knockdown in vitro and in vivo. **a** Western blot analysis verified vshRNA-mediated interference of USP7 expression in H460 cells; **b** growth curves of H460-vshUSP7 cells compared with control cells using the CCK-8 assay; **c** representative inhibition of foci formation in monolayer culture due to the interference of USP7 expression; **d**, **e** representative tumors formed in nude mice by control cells and H460-vshUSP7 cells; **f**, **g** qRT-PCR and Western blot were conducted to confirm the expression of USP7 in H460 generated xenografts

To evaluate the effect of USP7 expression on tumorigenesis, USP7 expression was knocked down in H460 cells (a large cell lung cancer cell line) using shRNAs (cells termed H460-shUSP7). The shUSP7 2# was the most effective knockout sequence and used for further experiments (Fig. 2a). Compared with control cells, cells with an interference of USP7 effectively inhibited cell tumorigenesis, including cell growth rate and foci formation frequency in vitro assays (Fig. 2b, c).

To further explore the tumorigenic ability of USP7 in vivo, tumor formation in nude mice was conducted by subcutaneously injecting H460-shUSP7 cells (H460-vec cells as controls) into five nude mice. The results showed that tumor formation in nude mice injected with H460-shUSP7 cells was significantly suppressed (Fig. 2d, e). With qRT-PCR and Western blot, we confirmed that USP7 was expressed higher in H460-vec cells-derived tumors (Fig. 2f, g). These results strongly suggest that USP7 plays vital roles in promoting tumor development.

### USP7 downregulation promotes lung cancer cell apoptosis

**Fig. 3:**
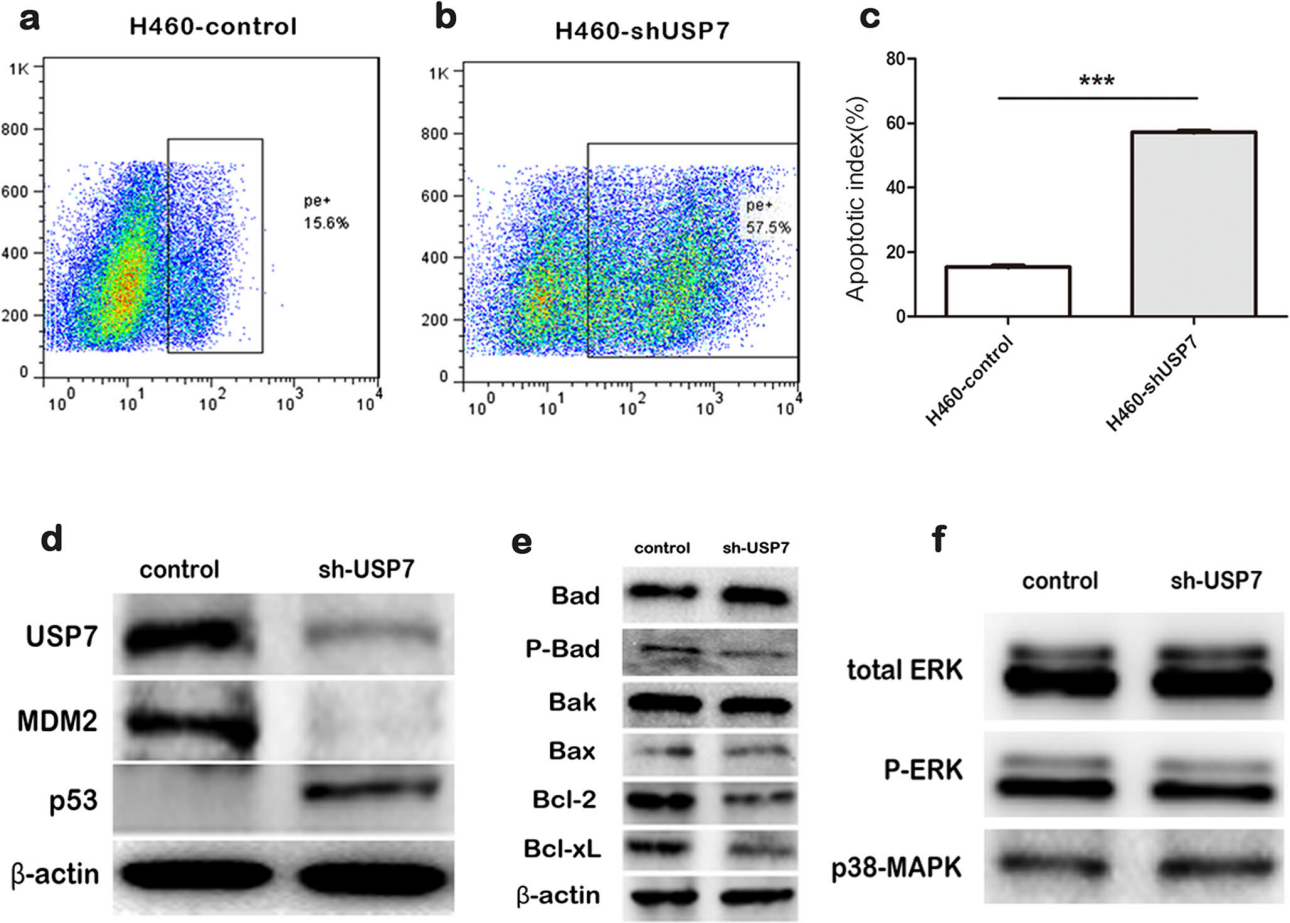
USP7 downregulation promotes lung cancer cells apoptosis. **a**–**c** Analysis of H460-vshUSP7 and control cell apoptosis using the Annexin V-PE apoptosis detection kit; **d** the interference of USP7 expression downregulated MDM2 protein expression but upregulated p53 protein levels; **e** the interference of USP7 expression suppressed the anti-apoptotic proteins Bcl-2, Bcl-xL, and phosphorylated Bad; **f** a comparison of ERK, P-ERK, and p38-MAPK protein expression between H460-vshUSP7 and control cells by Western blot analysis

USP7 reportedly regulates the apoptosis process [11]. To investigate the effect of USP7 expression on apoptosis in NSCLC cells, annexin V-PE was used to label apoptotic cells. We found that H460-shUSP7 cells contained a significantly greater number of apoptotic cells than control cells (Fig. 3a–c). Apoptosis is often triggered by p53 [12], and previous research has confirmed that p53 is regulated by USP7 through the oncogene MDM2 [13–15]. However, the role of USP7 in the p53-MDM2 pathway is currently controversial [14–16]. Consequently, we investigated p53 and MDM2 expression differences between H460-control and H460-shUSP7 cells. The results showed that p53 was dramatically increased in H460-shUSP7 compared to H460-control cells. However, MDM2 expression was downregulated when USP7 expression was knocked down (Fig. 3d).

To elucidate the molecular basis of apoptosis inhibition mediated by USP7, we further analyzed the effects of USP7 on the Bcl-2 protein family (mitochondrial proteins critical for performing cell apoptosis). We found that the levels of Bcl-2, Bcl-xL, and phosphorylated Bad were significantly downregulated after USP7 expression was knocked down (Fig. 3e). As Bad phosphorylation is promoted through the ERK and p38-MAPK pathways [17, 18], we examined these signaling pathways by Western blot analysis. However, the results showed no significant changes in the phosphorylation levels of ERK and p38-MAPK (Fig. 3f). The results indicated that overexpression of USP7 mediated apoptosis by regulating the p53-MDM2 pathway, which resulted in the upregulation of the Bad phosphorylation.

### USP7 expression affects NSCLC cell invasiveness and cells EMT

**Fig. 4:**
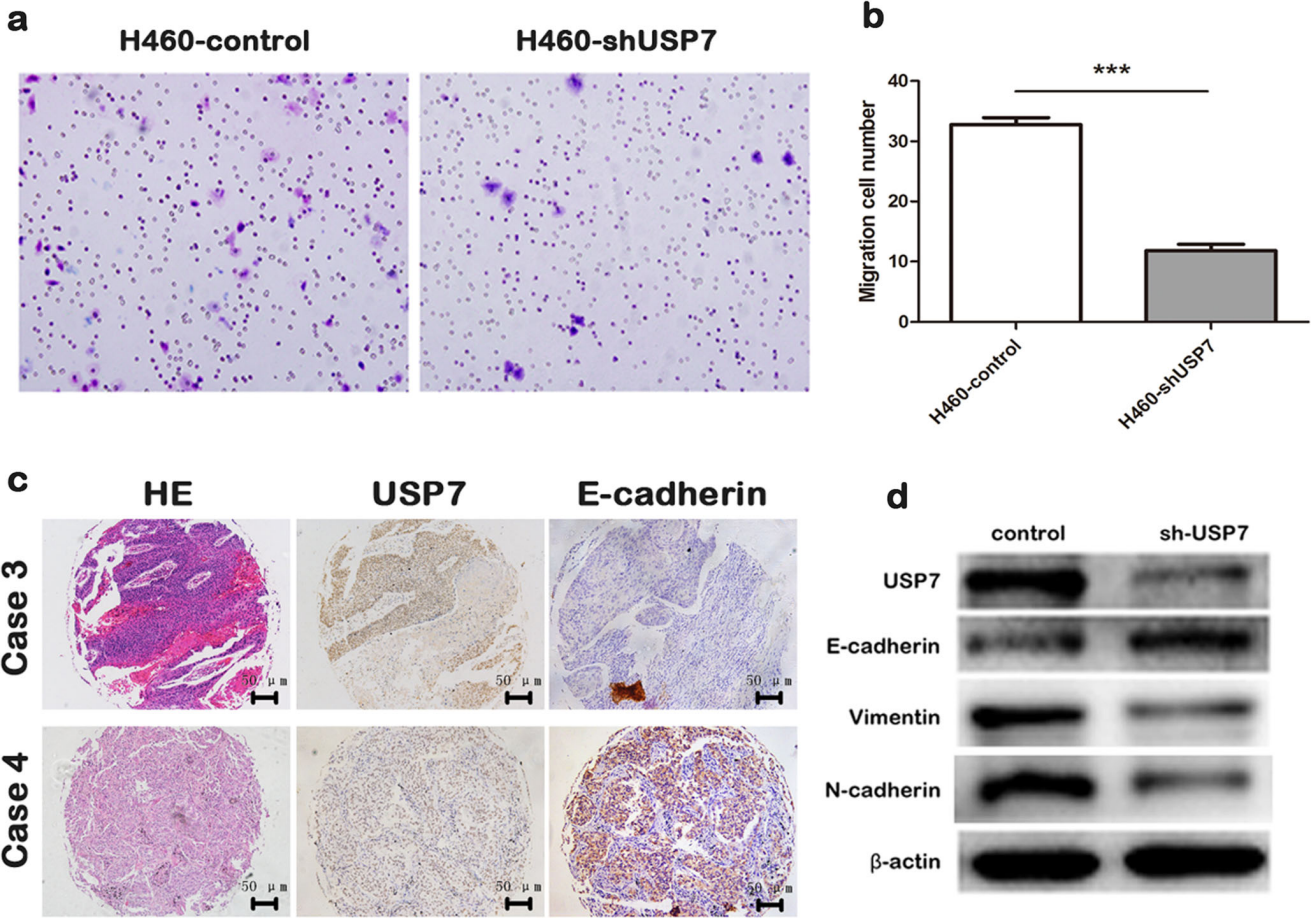
USP7 expression affects NSCLC cell invasiveness and cells EMT. **a**, **b** The interference of USP7 expression inhibited H460 cell invasion; **c** high level of USP7 was more often with the low expression of E-cadherin in cancer tissues; **d** the interference of USP7 expression upregulated the expression of E-cadherin, but downregulated vimentin and N-cadherin expression

To investigate the impact of USP7 on cancer cell invasiveness, we used transwell assays to study the different invasive abilities between H460-shUSP7 and H460-control cells. The knockdown of USP7 significantly inhibited cell invasiveness (Fig. 4a, b).

Epithelial-mesenchymal transition (EMT) plays a decisive role in cancer progression and metastasis [19]. We therefore analyzed the relationship between the USP7 and epithelial and mesenchymal markers expression in cancer tissues and cells. Firstly, we found that the high level of USP7 is more often with the low expression of E-cadherin in cancer tissues (Fig. 4c). Consistently, we observed an upregulation in E-cadherin protein levels, but a downregulation in Vimentin and N-cadherin proteins levels, after USP7 knockdown by shRNAs (Fig. 4d). These results indicated that USP7 potentially participates in EMT to promote NSCLC cell invasiveness.

## Discussion

Considerable research has found that USP7 is critical for controlling cell death and proliferation, and regulates key biological signaling pathways in tumorigenesis [5, 6, 11, 19, 20]. Although, the reduction of USP7 combining with p53 gene status has been reported to be a significant indicator of poor prognosis in adenocarcinoma patients, the roles of USP7 in lung squamous cell carcinoma and large cell carcinoma are largely unknown. Here, our results indicate that lung squamous cell carcinoma and large cell carcinoma tumors and NSCLC cell lines express high levels of USP7 compared with corresponding non-tumorous tissues or immortalized normal lung cell lines. Clinically, large tumor size, advanced tumor stage, and lymph node metastasis were associated with elevated USP7 expression. Furthermore, Kaplan-Meier analysis revealed that the patients with high USP7 levels had significantly shorter overall survival than those with low USP7 expression in 110 squamous cell carcinoma and large cell carcinoma patients. By knocking down USP7 expression with shRNA interference, we revealed that lowering USP7 expression could effectively suppress cell growth rate, foci formation in vitro, and tumor formation in vitro and in vivo. Thus, we showed that high USP7 levels promote squamous cell carcinoma and large cell carcinoma progression.

USP7 is known to regulate the p53-MDM2 pathway [7, 11, 13]. MDM2 is a major negative regulator of p53 [21], and recent studies have found that p53 and MDM2 both bind to the N-terminal TRAF-like domain of USP7 in a mutually exclusive manner [13, 22]. Our data showed dramatic increases in p53 expression and decreases in MDM2 expression in USP7 knockdown H460 cells compared with control cells. This suggests USP7 may negatively regulate p53 levels through MDM2. p53 is a key protein in mitochondrial apoptotic pathways [12, 23], and our research found that the knockdown of USP7 expression in H460 cells can promote apoptosis, again suggesting that USP7 participates in the apoptotic pathway.

In the mitochondrial apoptotic pathway, Bcl-2 family apoptotic-modulating proteins are closely regulated by p53 [12, 24]. Our data showed that the knockdown of USP7 is associated with the downregulation of Bcl-2 and Bcl-xL anti-apoptotic proteins. As p53 can antagonize the anti-apoptotic effects of Bcl-2 and Bcl-xL [12], USP7 may inhibit the Bcl-2 and Bcl-xL anti-apoptotic effect by negatively regulating p53. Interestingly, the level of phosphorylated Bad was also downregulated by USP7 knockdown, and dephosphorylated Bad can interact with Bcl-2 and Bcl-xL in mitochondria, thereby inactivating these anti-apoptotic proteins and inducing apoptosis [25]. Bad phosphorylation is reportedly promoted through the ERK and p38-MAPK pathways [24]. Therefore, as ERK and p38-MAPK phosphorylation levels did not significantly change after USP7 knockdown in NSCLC cells, USP7 may directly affect the phosphorylation status of Bad.

We found that USP7 knockdown significantly inhibited cell invasiveness. Furthermore, upregulation in E-cadherin protein levels, but downregulation in Vimentin and N-cadherin proteins levels, was found after the knockdown in USP7 levels. These results indicate that USP7 may participate in EMT to promote the invasive ability of cancer cells.

Overall, we have demonstrated that lung squamous cell carcinoma and large cell carcinoma overexpressed USP7, and high levels of USP7 play an important role in tumor invasion and metastasis in squamous cell carcinoma and large cell carcinoma. USP7 is a novel marker for predicting the prognosis of patients with lung squamous cell carcinoma and large cell carcinoma, and may serve as a potential therapeutic target.

## Electronic supplementary material

Below is the link to the electronic supplementary material.

Supplementary Fig. 1 The expression of USP7 in USP7^low^ tumor tissues and their non-tumorous tissues. Case 5 and case 6: the USP7 expression was lower in NSCLC tissues compared to non-tumorous samples; case 7 and case 8: the USP7 expression was slight higher in NSCLC tissues compared to non-tumorous samples. (GIF 823 kb)

High Resolution Image (TIFF 13304 kb)

Supplementary Table 1 (DOC 27 kb)

## Abbreviations

NSCLC: Non-small cell lung cancer
qRT-PCR: Quantitative real-time polymerase chain reaction
MDM2: Murine double minute
HSV1: Herpes simplex virus type 1
EBNA1: Epstein-Barr nuclear antigen 1
DMEM: Dulbecco Modified Eagle medium
TNM: Tumor/lymph node/metastasis
OS: Overall survival
TMA: Tissue microarray
EMT: Epithelial-mesenchymal transition
