# Supplementary figures and images for: USP7 overexpression predicts a poor prognosis in lung squamous cell carcinoma and large cell carcinoma
Source: Tumour Biol. 2014 Dec 18;36(3):1721–9. doi: 10.1007/s13277-014-2773-4 (PMC4375295; doi:10.1007/s13277-014-2773-4)

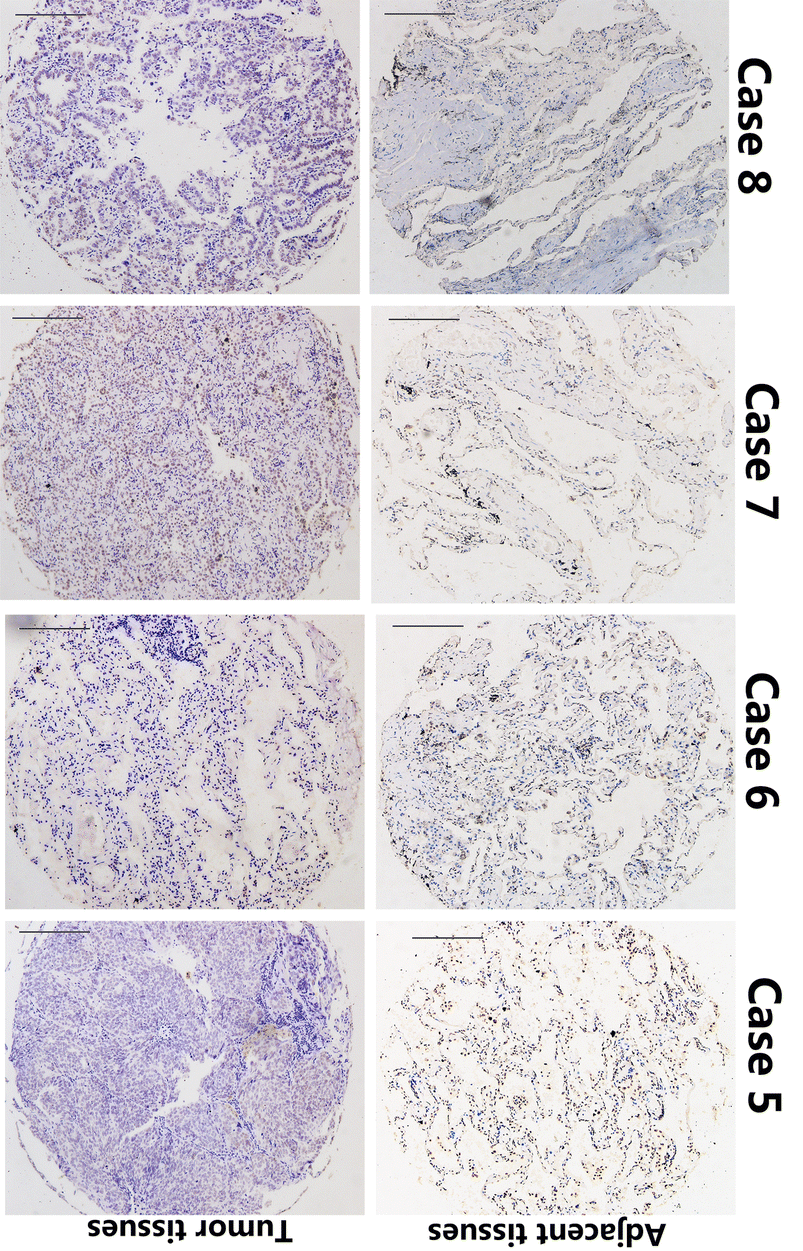

Supplement: Supplementary file 1 — The expression of USP7 in USP7low tumor tissues and their non-tumorous tissues. Case 5 and case 6: the USP7 expression was lower in NSCLC tissues compared to non-tumorous samples; case 7 and case 8: the USP7 expression was slight higher in NSCLC tissues compared to non-tumorous samples. (GIF 823 kb) [file 13277_2014_2773_Fig5_ESM.gif]

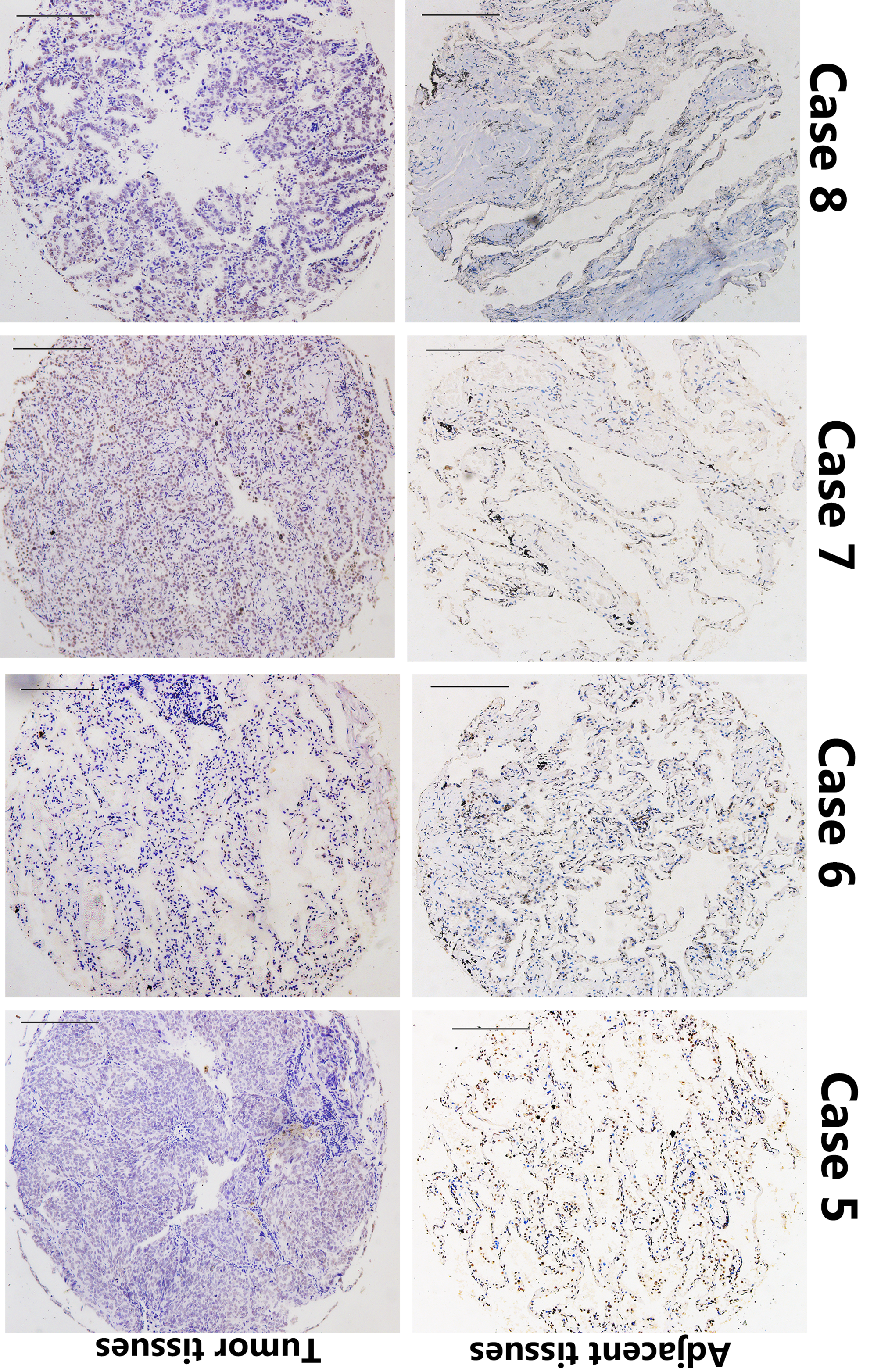

Supplement: Supplementary file 2 — High Resolution Image (TIFF 13304 kb) [file 13277_2014_2773_MOESM1_ESM.tif]
