# Supplementary material for: USP7 overexpression predicts a poor prognosis in lung squamous cell carcinoma and large cell carcinoma
Source: Tumour Biol. 2014 Dec 18;36(3):1721–9. doi: 10.1007/s13277-014-2773-4 (PMC4375295; doi:10.1007/s13277-014-2773-4)
Supplement: Supplementary file 3 — (DOC 27 kb) [file 13277_2014_2773_MOESM2_ESM.doc]

**Supplementary Table 1 The expression of USP7 in** USP7^low^ tumor tissues and their non-tumorous tissues

| **USP7 expression**  （Tumor tissues *versus* Non-tumorous tissues） | **No. of patient** |
| --- | --- |
| Lower | 41 |
| Slight higher | 12 |
